# Supplementary figures and images for: Mating suppresses sperm-dependent male avoidance in C. elegans hermaphrodites
Source: PLoS One. 2026 Jan 27;21(1):e0339893. doi: 10.1371/journal.pone.0339893 (PMC12843523; doi:10.1371/journal.pone.0339893)

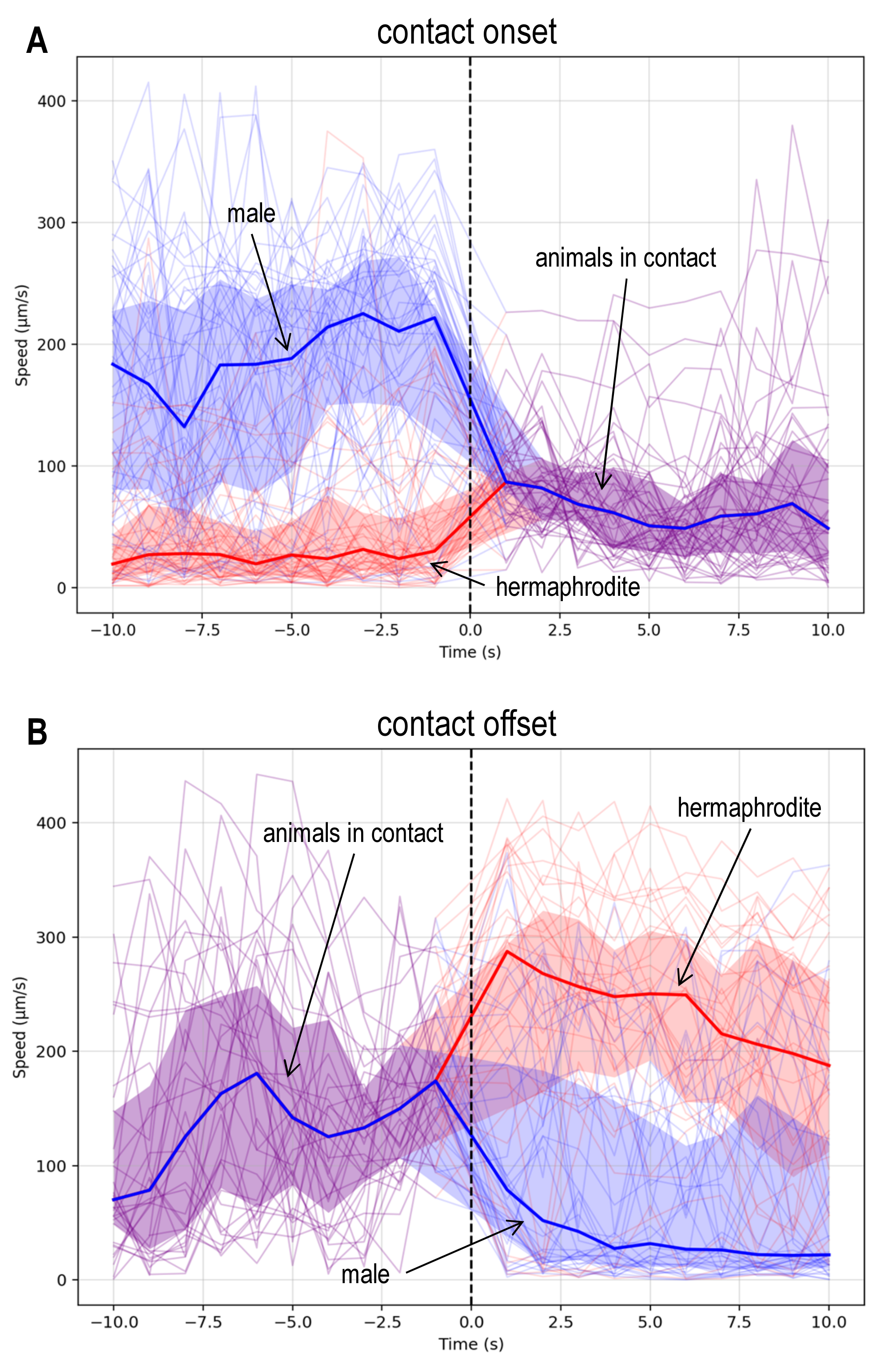

Supplement: S1 Fig — Median locomotion speeds of hermaphrodites (red) and males (blue) aligned to contact onset (A) or offset (B). At contact onset, the hermaphrodite’s speed increased (p = 1.4 x 10^−5^) while the male’s speed decreased (p = 6.9 x 10^−8^). At contact offset, the hermaphrodite’s speed again increased (p = 0.0024) while the male’s speed decreased (p = 5.8 x 10^−4^). Shaded areas show the interquartile range and individual worms are shown as faint lines. (TIF) [file pone.0339893.s001.tif]
